# Selenium Transport Mechanism via Selenoprotein P—Its Physiological Role and Related Diseases

**DOI:** 10.3389/fnut.2021.685517

**Published:** 2021-05-28

**Authors:** Yoshiro Saito

**Affiliations:** Laboratory of Molecular Biology and Metabolism, Graduate School of Pharmaceutical Sciences, Tohoku University, Sendai, Japan

**Keywords:** low-density lipoprotein receptor-related protein, lysozome, selenoprotein synthesis, ApoER2, LRP1, megalin, selenium transport system

## Abstract

Selenoprotein P (SELENOP) is selenium (Se)-containing protein in plasma, which is primarily produced in the liver. The “P” in SELENOP originated from the presence in plasma. SELENOP contains selenocysteine, a cysteine analog containing Se instead of sulfur. SELENOP is a multi-functional protein to reduce phospholipid hydroperoxides and to deliver Se from the liver to other tissues, such as those of the brain and testis, playing a pivotal role in Se metabolism and antioxidative defense. Decrease in SELENOP causes various dysfunctions related to Se deficiency and oxidative stress, while excessive SELENOP causes insulin resistance. This review focuses on the Se transport system of SELENOP, particularly its molecular mechanism and physiological role in Se metabolism. Furthermore, the chemical form of Se and its biological meaning is discussed.

## Introduction

Selenoprotein P (encoded as SELENOP) was first described in 1973, and its character was reported as the major selenium (Se)-containing protein in plasma (1, 2). SELENOP is mainly synthesized in the liver and secreted to the plasma after cleavage of the signal peptide. The “P” in SELENOP denotes its presence in plasma. SELENOP contains the essential trace element Se in the form of selenocysteine (Sec), which is an analog of cysteine that contains Se instead of sulfur (2–4). Twenty-five genes encoding human Sec-containing proteins, i.e., selenoproteins, have been discovered, which play significant roles in several physiological processes such as antioxidant defense and metabolism; five types of glutathione (GSH) peroxidases (GPXs) play significant roles in the removal of several hydroperoxides, three types of thioredoxin reductases (TRXRs) in redox regulation, three types of iodothyronine deiodinases in the regulation of thyroid hormones, and selenophosphate synthetase 2 (SEPHS2) in Sec synthesis (4, 5). Most selenoproteins have a Sec residue, while SELENOP has 10, making it multifunctional (6, 7). SELENOP possesses two different functions: Se transport activity to supply Se to cells and GPX-like activity to reduce phospholipid hydroperoxide (8, 9). SELENOP maintains selenoenzymes in several tissues and plays a crucial role in antioxidative defense and Se metabolism (10, 11). A decrease in SELENOP causes deficiency in selenoproteins and various dysfunctions with oxidative stress, while excess SELENOP induces insulin resistance, which can lead to type 2 diabetes (12, 13). This review focuses on the Se transport system via SELENOP, particularly its molecular mechanism and physiological role in Se metabolism. Furthermore, the chemical form of Se and its biological meaning are discussed.

## Selenoproteins in Human Plasma

**Figure 1:**
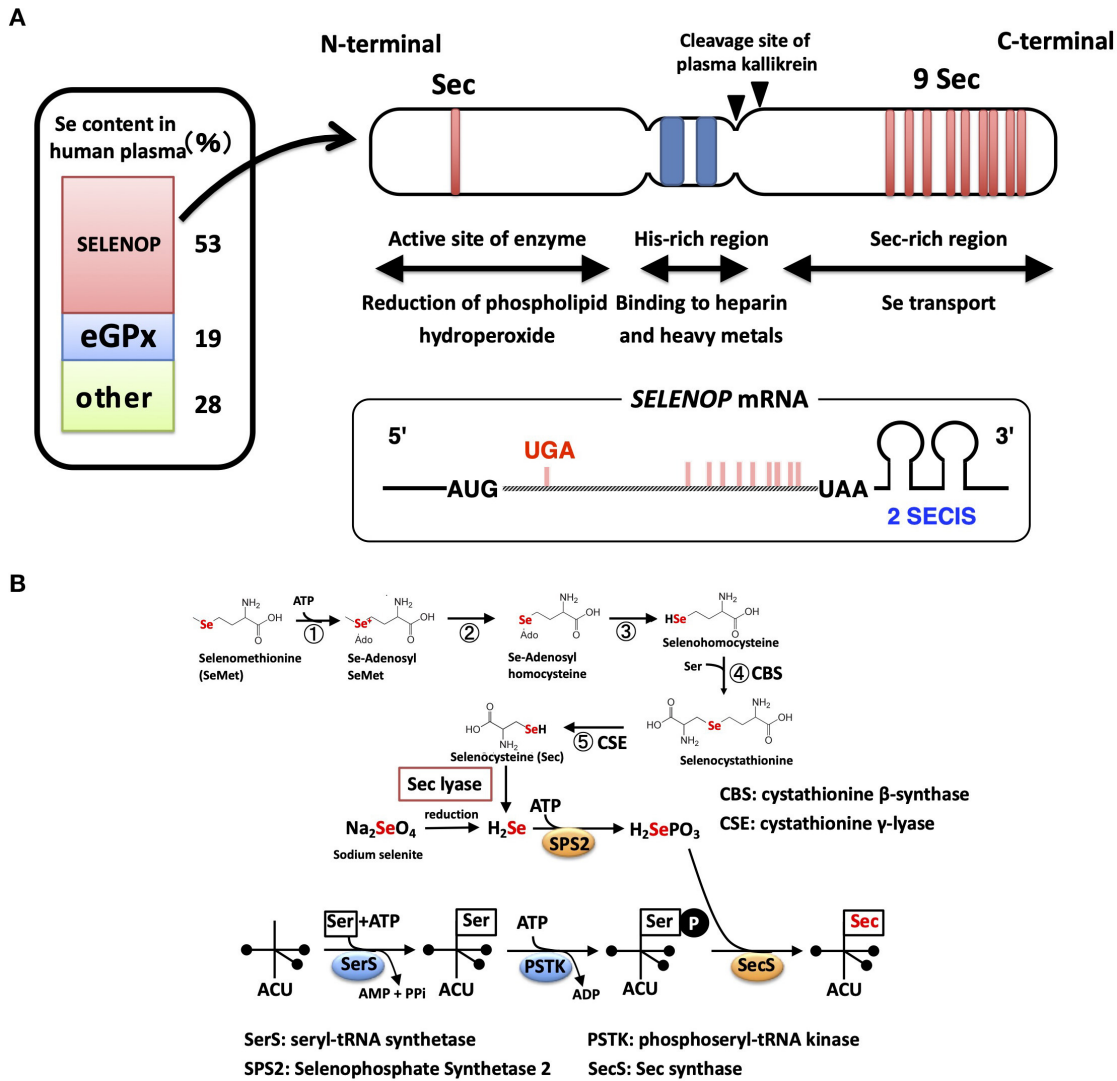
Structure of the SELENOP and selenocysteine synthesis pathway. **(A)** Domain structure of human SELENOP. The Se content estimated from SELENOP- and eGPX-deficient human plasma is shown in the left panel. **(B)** Selenocysteine synthesis pathway. SeMet is converted to Sec with Met-metabolizing enzymes without discrimination with Met. Inorganic Se is produced with Sec lyase and used for the synthesis of Sec.

In human plasma, there are two kinds of selenoproteins: SELENOP and extracellular GPX (GPX3), possessing Se as Sec residue (14). SELENOP is primarily secreted from the liver, while GPX3 is synthesized in the kidney. To estimate the Se content derived from each selenoprotein, SELENOP- and GPX3-deficient human plasma were prepared using immobilized specific antibody (9). The absorption of SELENOP resulted in the decrease in Se content to 47% of the total, while the removal of GPX3 decreased it to 81%, indicating that 53 and 19% of plasma Se is derived from SELENOP and GPX3, respectively (Figure 1). Similar results on the contributions of SELENOP and GPX3 to plasma Se have been reported by the laboratories of Burk and Schomburg (10), Olson et al. (15), and Brodin et al. (16). The residual 28% of Se might be derived from selenomethionine (SeMet) in albumin, and/or low molecular Se compounds, which have in part been identified as selenosugars (17).

Sec is encoded by the UGA codon, known as a stop (opal) codon, and is called the 21st amino acid in the genetic code (18, 19). In eukaryotes, the Sec insertion sequence (SECIS), which is a specific hairpin structure located in the 3′ untranslated region (3′UTR) of selenoprotein mRNA, is essential for the incorporation of Sec during the biosynthesis of selenoproteins. SECIS binds SECIS-binding protein 2 (SBP2) and forms a complex for Sec incorporation via the recruitment of the Sec-specific eukaryotic elongation factor (eEFsec) and Sec-tRNA^Sec^ (an anticodon complementary to the UGA codon) (20).

The mRNA of SELENOP has a unique property of containing 10 UGA codons in the open reading frame (ORF) and two SECIS in the 3′UTR, while other selenoprotein mRNAs have only one SECIS element (21) (Figure 1A). Multiple Sec residues in SELENOP are important for its function; one N-terminal Sec residue forms an active site of enzyme activity to reduce phospholipid hydroperoxide, while the nine C-terminal Sec residues function as Se transporter (22) (Figure 1A). The first SECIS element, which is located on the 5′ side near the stop codon, mainly facilitates the processive Sec incorporation, while the second SECIS functions slow decoding at the first UGA codon (23). Plasma kallikrein cleaves SELENOP by limited proteolysis with Arg-235–Gln-236 and Arg-242–Asp-243, generating N-terminal fragments (residues 1–235) with enzyme activity and C-terminal fragment (residues 243–361) exhibiting Se-supply activity (22). N-terminal, a possible catalytic center of SELENOP, has U(Sec)XXC motif, similar to the active-site of thioredoxin (CXXC motif), which suggests the reactivity of SELENOP against protein thiols. Actually, SELENOP has broad thiol specificity (24), and it uses not only GSH but also other thiols, such as TRX, dithiothreitol, and mercaptoethanol, as reducing agents, while the thiol specificity of cellular GPX (GPX1) is narrow, using only GSH as reductant. In human plasma, the concentration of GSH and TRX is ~5 μM and 2 nM, respectively (25, 26). Based on kinetic analysis, SELENOP uses TRX 500-fold more effectively than GSH (24), but it is still uncertain whether both thiols could contribute to the reduction of SELENOP in plasma.

The mRNA of GPX3 has a UGA codon in the ORF and a SECIS in the 3′UTR, which is a representative feature of selenoproteins, such as the GPX family. GPX3 has catalytic triad composed of Sec, Gln, and Trp, in which the Se of a Sec residue is activated by hydrogen bonding to Trp and Gln residue (27). GPX3, like GPX1, is a homotetramer, while GPX4 (PHGPX) and SELENOP are monomers. GPX3 reduces diverse hydroperoxides, including hydrogen peroxide, tert-butyl hydroperoxide, and phospholipid hydroperoxide (24). GPX3 also possesses broad thiol specificity and uses TRX, dithiothreitol, and mercaptoethanol as reducing agents.

Se incorporated in Sec is specifically regulated via the synthesis of selenoproteins (28, 29). Sec is synthesized on tRNA using inorganic Se (Figure 1B). Seryl-tRNA synthetase (SerRS) binds Ser to tRNA^Sec^, which has an anticodon of UGA, and the hydroxyl residue of Ser undergoes phosphorylation. SEPHS2 produces selenophosphate (H_2_SePO_3_) from inorganic Se and ATP. SEPHS2 is a selenoprotein, suggesting the self-regulation of the Sec synthesis system. The Sec synthase catalyzes the formation of a selenol residue on tRNA using H_2_SePO_3_ and phosphorylated Ser-tRNA^Sec^ (Figure 1B). Inorganic Se, such as sodium selenite, is recognized as “Se” in mammals and is reduced, phosphorylated, and incorporated into the synthesis pathway of Sec. Sec derived from the diet is also recognized as “Se” and is converted to inorganic Se by Sec lyase (30) (Figure 1B). In contrast, SeMet, a methionine analog that contains Se instead of sulfur, is recognized as “Met” in mammals and is incorporated into proteins in the same manner as Met (31). SeMet is also metabolized by Met-metabolizing enzymes. Se in SeMet is recognized as “Se” when it is converted to Sec via metabolism by cystathionine β-synthase and cystathionine γ-lyase, which are also known as Cys-persulfide-producing enzymes (Figure 1B). SeMet is considered “masked Se,” and the contents of SeMet in the diet influence the concentration of Se in blood. As described above, ~70% of “Se” is derived from Sec of SELENOP and GPX3, and the other 28% might be from SeMet and low molecular weight Se. Sec lyase is specific for Sec and provides “Se” to SEPHS2. Only through this pathway, Se is used for the synthesis of Sec on the tRNA^Sec^.

## Se Transport System Via Selenoprotein P and Others

**Figure 2:**
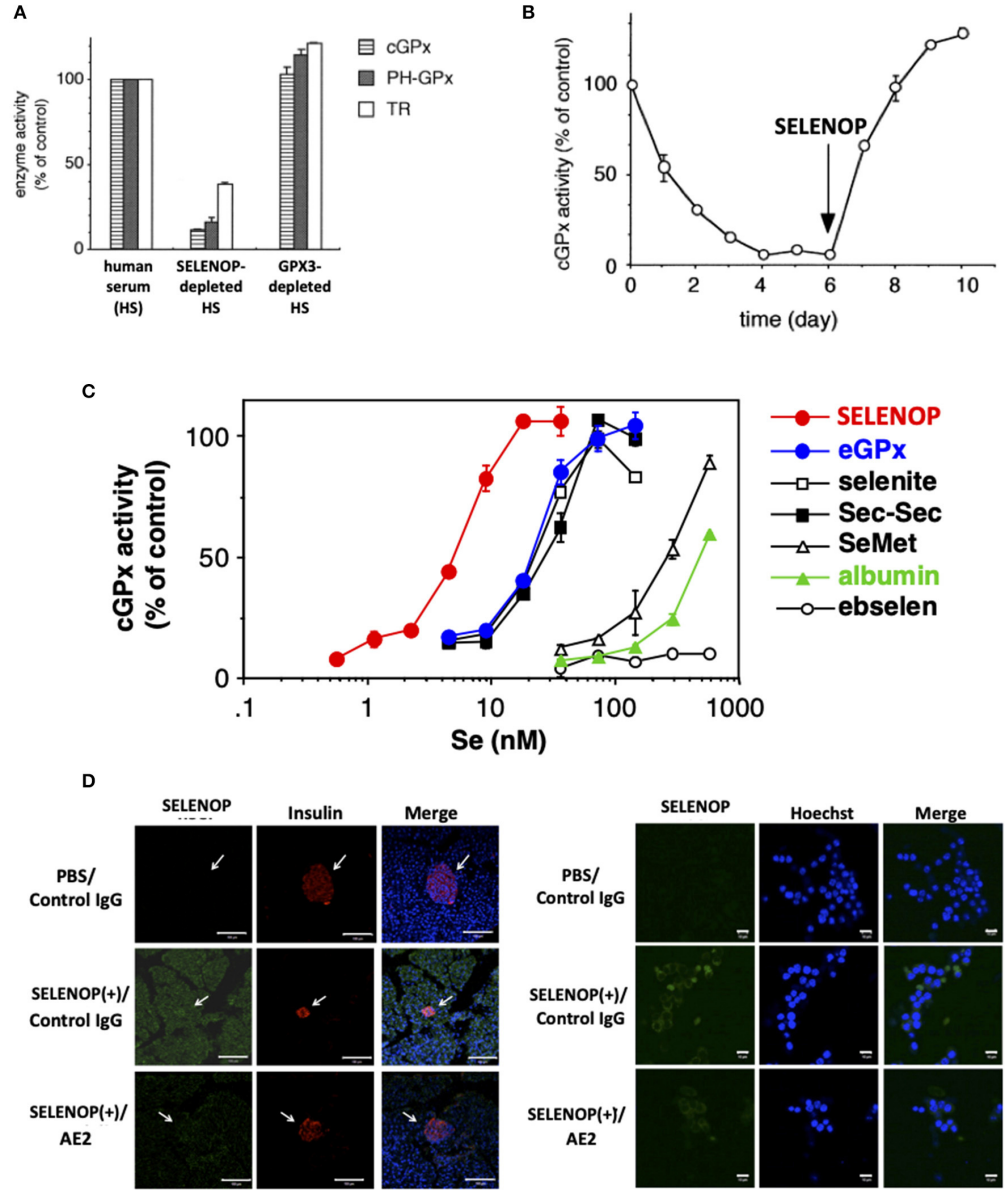
Se transport activity of SELENOP. **(A)** Effect of serum selenoprotein depletion on cellular selenoenzyme activities. The Jurkat cells were cultured for 3 days in a medium containing each human serum. **(B)** Effect of SELENOP on cGPX activity. The Jurkat cells were cultured in a medium containing SELENOP-depleted human serum, and cGPX activity was measured. After 6 days, purified SELENOP was added. **(C)** Effect of the addition of Se-containing materials on cGPX activity. In the presence of variable amounts of Se-containing materials, the Jurkat cells were cultured with SELENOP-depleted serum. **(D)** Immunohistochemical analysis of incorporated human SELENOP. Left panel. SELENOP-neutralizing monoclonal antibody (mAb) AE2 improved pancreatic β-cell area. Pancreas tissues from neutralizing mAbAE2- and human SELENOP-treated mice were examined immunohistochemically using anti-insulin Ab (indicative of β-cells) and anti-human SELENOP Ab. Cell nuclei were stained with DAPI (blue). The distribution of human SELENOP in pancreatic β-cells was decreased in mice administered with AE2. Right panel. MIN6 cells were incubated with 10 μg/ml human SELENOP in the presence of AE2 and control IgG (500 μg/ml) for 48 h. Treated MIN6 cells were examined immunohistochemically using anti-human SELENOP Ab. [**A–C**, (9) with permission and modifications: 5051220115395, D, reference (34) with permission:CC-BY].

Serum free-culture of neurons and several cells requires Se addition, because SELENOP functions as a Se carrier in serum-containing culture (32, 33). SELENOP was identified as a survival-promoting factor for cultured neurons in serum-free medium in 1998, suggesting the Se transport system via SELENOP (32). The Se transport system of SELENOP has been described in *in vitro* experiments using human T lymphoma Jurkat cells and selenoprotein-deficient human serum prepared by the immobilized antibody for SELENOP and GPX3 (9). When cultured with SELENOP-depleted serum, and not GPX3-depleted or the control serum, the activity of GPX1 decreased to 17% compared with that of the control (Figure 2A). The activity of two other selenoproteins, GPX4 and TRXR, also decreased to 16 and 38%, respectively (9). When cultured with SELENOP-depleted serum, the whole-cell Se content also decreased to 19% compared with that of control cells. Time-dependent analysis revealed that the GPX1 activity of cells cultured with SELENOP-depleted serum was almost undetectable after 4 days (Figure 2B). The addition of 270 ng/ml purified SELENOP (SELENOP concentration of 5% human serum) resulted in the complete recovery of GPX1 activity. Thus, SELENOP functions as a major Se transporter in this culture system.

The comparison of SELENOP with other Se-containing materials as a Se supplier demonstrated that SELENOP was the most effective with a 50% effective dose (ED_50_) of 5 nM (Se equivalent), followed by GPX3, sodium selenite, selenocystine, SeMet, and albumin (9) (Figure 2C). The ED_50_ of the former three reagents was 25 nM, and that of the latter two was 300 and 500 nM, respectively. Ebselen had no effect up to 500 nM. These results suggest the effective Se transport system via SELENOP (9). Next to SELENOP, the inorganic Se and Sec group, which is recognized as “Se,” is also a good source of Se. Third group SeMet and albumin, which is classified as “masked Se,” is not effective, but it could function as a Se source in high concentration. The Se concentrations of human plasma used in the previous study was 1.5 μM, and the estimated Se concentration of SELENOP, GPX3, and the others were 750, 300, and 450 nM, respectively. Thus, it is considered that the effective Se transport system of SELENOP might be less functional in cells that have direct contact with plasma, and that it will be effective in the interstitial fluid where the plasma is diluted. It needs further characterization in the SELENOP concentration of the interstitial fluid. The biological significance of the effective Se transport system via SELENOP has been demonstrated by SELENOP KO mice (35, 36). The decrease in tissue Se in SELENOP KO mice has been reported in the brain, kidney, testis, and bone (35–38). The severe phenotype of SELENOP is spermatogenesis disorder, which could not be recovered by Se supplementation, while the other disorders might be partly recovered by Se supplementation, suggesting the compensatory role of the Se transport system other than SELENOP. It is also notable that SELENOP is contained in mothers' milk and has the function to supply Se to offspring (37). The effective Se transport system of SELENOP is mediated by its receptors, such as apolipoprotein E receptor 2 (ApoER2), megalin, and low-density lipoprotein receptor-related protein 1 (LRP1), which belong to lipoprotein receptors. In addition, Se transport of SELENOP has been observed in a receptor-independent manner (pinocytosis) (39).

## The Receptors for Selenoprotein P

Three kinds of SELENOP receptors have been identified, namely, ApoER2 (LRP8), megalin (LRP2), and LRP1. ApoER2 and megalin were first discovered by immobilized SELENOP affinity column chromatography (40, 41), while LRP1 was identified by the siRNA experiment on C2C12 myocytes based on the expression of LRPs (42). SELENOP transports Se to several tissues via these receptors. Based on the phenotype of each receptor KO mice, ApoER2 is associated with SELENOP uptake in the brain, testis, and bone; megalin with the kidney and brain; and LRP1 with the skeletal muscle. It has been reported that megalin mediates brain Se uptake and that Apoer2 mediates neuronal SELENOP uptake (43).

Similar phenotypes of SELENOP KO mice have been reported in the brain and testis of ApoER2 KO mice, indicating the biological significance of receptor-mediated uptake of Se in these tissues (35–37, 44). Similar phenotypes in the brain and testis have been reported in SELENOP^Δ240−361^ mice, in which the Sec-rich C-terminal domain of SELENOP had been deleted (34). The interaction between the C-terminal domain of SELENOP and the YWTD β-propeller domain of ApoER2 has been reported, and the importance of this interaction, particularly in maintaining Se levels in the brain and testis, has been manifested in the phenotypes of these KO mice (45). In the normal diet that contains enough Se (0.4 mg Se/kg diet), Se levels in other tissues such as the intestine and lung of SELENOP KO mice, except for the brain, testis, and bone, do not differ greatly from those in WT mice. Selenite in mouse chow is not a normal constituent of diet, but can be imported directly or after metabolism into SELENOP- and ApoER2-deficient cells. In the case of the brain of SELENOP KO mice, Se content was greatly affected by diet; 0.1 mg Se/kg diet induced a significant decrease in brain Se (~50% of WT) and caused severe motor dysfunction, which needed humane endpoint (44). These observations suggest the role of the Se transport system via not only SELENOP but also SELENOP-independent systems *in vivo*. These observations indicate the role of SELENOP in the Se transport system, which is particularly effective to maintain homeostasis under the Se-deficient condition. This system might be significant to survive in the evolutionary process.

Megalin plays a crucial role in the reabsorption of SELENOP in the kidney, and increase in urine SELENOP has been shown in megalin KO mice (40, 43). Megalin is a large glycoprotein (~600 kDa), which possesses four large clusters of ligand-binding repeats stabilized with a disulfide bond. In the kidney, proximal tubule epithelial cells highly express megalin, indicating the physiological role of megalin in the reabsorption of SELENOP. LRP1 is identified as SELENOP receptor in the skeletal muscle (42). LRP1 is a super-macromolecule with a size of about 600 kDa and has a diverse set of ligands, such as amyloid β. Skeletal muscle has relatively low affinity for SELENOP, and the Se content does not change in SELENOP KO mice. However, the increase in SELENOP in the case of type 2 diabetes enhances the uptake of SELENOP via LRP1, which is related to the increase in insulin and exercise resistance (42). Thus, the SELENOP–LRP1 axis is an important therapeutic target for the cure of type 2 diabetes. Human embryo rhabdomyosarcoma RD cells express both ApoER2 and LRP1, which show low affinity for SELENOP uptake, and high amount of SELENOP is necessary for the uptake and use of Se in SELENOP (46). Treatment of RD cells with siRNA for either ApoER2 or LRP1 resulted in significant decrease in SELENOP uptake and increase in GPX1, suggesting both receptors coordinately work for SELENOP uptake. Diverse variants of ApoER2 have been known, while the relationship between ApoER2 variants and SELENOP uptake is unknown.

## Se Transport System Via Selenoprotein P

SELENOP possesses Se as Sec, which is covalently bonded to the polypeptide chain. To use the Se in SELENOP for selenoprotein synthesis, several biochemical steps are necessary. Based on previous reports, it has considered that SELENOP is incorporated into the cell, and then is degraded to amino acids in the lysosome (45). It is notable that the life span of incorporated SELENOP is long, which makes it possible to observe incorporated cellular SELENOP by *in vivo* and *in vitro* immunostaining (46) (Figure 2D). SELENOP is a glycoprotein, and the carbohydrate chains in SELENOP might function to prevent proteolysis in lysosomes. The generated Sec from SELENOP is cleaved with Sec lyase, converting to hydrogen selenide H_2_Se, which is further phosphorylated to H_2_SePO_3_ with SEPHS2, as described above. The toxicity level of H_2_Se is high, and the phosphorylation of H_2_Se is important to prevent its toxicity. Notably, the necessity for Sec lyase in Se transport of SELENOP is not clear, because mice lacking Sec lyase do not have selenium deficient phenotypes, such as male sterility (47).

SELENOP is synthesized in peripheral tissues and SELENOP-expressing cells are present in several tissues (48). Based on serum SELENOP concentrations of liver-specific SELENOP KO mice, 60% of SELENOP is estimated to be derived from the liver and the remaining 40% from other tissues (49). SELENOP expression in the brain is significant for maintaining Se and selenoprotein levels, and Se concentrations in the brain are preserved in liver-specific SELENOP KO mice. SELENOP expression has been reported in several cell types of the brain, such as neurons and ependymal cells, that are responsible for cerebrospinal fluid production (50). SELENOP synthesized in the brain is incorporated into other brain cells and used to synthesize selenoproteins, which help maintain Se concentrations in the brain. This system is called the SELENOP cycle, and it retains selenoproteins in several tissues and cells (51).

ApoER2 is a significant mediator of the SELENOP cycle. The similar phenotype between SELENOP and ApoER2 KO mice implies the role of ApoER2 in this cycle. Details are not fully elucidated, but the role of ApoER2 as a mediator of signal transduction has been known, which is realized by pulmonary arterial hypertension (PAH) where the increased expression of SELENOP in pulmonary artery smooth muscle cells (PASMCs) forms lesions (52). PAH-PASMCs are proliferative, and the pulmonary artery is constricted/occluded by the abnormal proliferation of PAH-PASMCs. The decrease in SELENOP expression by SELENOP-siRNA treatment inhibits the proliferation of PAH-PASMCs, and these effects are mediated by ApoER2 (52). Interestingly, the proliferative effects of SELENOP were not explained by Se transport activity; namely, the addition of selenocystine did not reproduce this effect of SELENOP, and proliferation-promoting effects were observed by the overexpression of the mutant in which all Secs were substituted with Cys. The proliferative effects of increased SELENOP on PAH-PASMCs are considered to be mediated by the cell signal from ApoER2 and HIF-1. Se-independent biological effects of SELENOP-ApoER2 axis have been described in a study on PAH, and it is interesting to speculate about the possibilities to relate to other physiological and/or pathological conditions.

## Conclusion

This review focuses on the Se transport system via SELENOP, particularly its molecular mechanism and role in Se metabolism. SELENOP is not a mere Se transporter. It plays the role of multifunctional protein to maintain cellular selenoproteins and regulate cellular redox homeostasis. Furthermore, the Se-independent role of SELENOP suggests the diverse biological and pathophysiological significance of this protein. Further research is necessary to understand the various roles of SELENOP.

## Author Contributions

The author confirms being the sole contributor of this work and has approved it for publication.

## Conflict of Interest

The author declares that the research was conducted in the absence of any commercial or financial relationships that could be construed as a potential conflict of interest.

## Abbreviations

ApoER2: apolipoprotein E receptor 2
GPX: glutathione peroxidase
GSH: glutathione
LRP1: low-density lipoprotein receptor-related protein 1
PAH: pulmonary arterial hypertension
PASMC: pulmonary artery smooth muscle cell
PHGPX: phospholipid hydroperoxide GPX
SBP2: SECIS-binding protein 2
Se: selenium
Sec: selenocysteine
SECIS: Sec insertion sequence
SeMet: selenomethionine
SELENOP: selenoprotein P
SEPHS2: selenophosphate synthetase 2
TRX: thioredoxin
TRXR: thioredoxin reductase
3′UTR: 3′ untranslated region.

## References

[1] Burk RF. Effect of dietary selenium level on Se binding to rat plasma proteins. Proc SocExpBiol Med. (1973) 143:719–22. 10.3181/00379727-143-37399 4719458

[2] Burk RF Hill KE. Selenoprotein P-expression, functions, and roles in mammals. BiochimBiophysActa. (2009) 1790:1441–7. 10.1016/j.bbagen.2009.03.026 19345254 PMC2763998

[3] Saito Y. Selenoprotein P as an *in vivo* redox regulator: disorders related to its deficiency and excess. J ClinBiochemNutr. (2020) 66:1–7. 10.3164/jcbn.19-31 32001950 PMC6983434

[4] Rayman MP. Selenium and human health. Lancet. (2012) 379:1256–68. 10.1016/S0140-6736(11)61452-9 22381456

[5] Labunskyy VM Hatfield DL Gladyshev VN. Selenoproteins: molecular pathways and physiological roles. PhysiolRev. (2014) 94:739–77. 10.1152/physrev.00039.2013 24987004 PMC4101630

[6] Schomburg L. Genetics and phenomics of selenoenzymes–how to identify an impaired biosynthesis? MolCell Endocrinol. (2010) 322:114–24. 10.1016/j.mce.2010.01.011 20083162

[7] Saito Y. Selenoprotein P as a significant regulator of pancreatic beta cell function. J Biochem. (2020) 167:119–24. 10.1093/jb/mvz061 31373634

[8] Saito Y Hayashi T Tanaka A Watanabe Y Suzuki M Saito E. Selenoprotein P in human plasma as an extracellular phospholipid hydroperoxide glutathione peroxidase. Isolation and enzymatic characterization of human selenoproteinP. J BiolChem. (1999) 274:2866–71. 10.1074/jbc.274.5.2866 9915822

[9] Saito Y Takahashi K. Characterization of selenoprotein P as a selenium supply protein. Eur J Biochem. (2002) 269:5746–51. 10.1046/j.1432-1033.2002.03298.x 12423375

[10] Burk RF Hill KE. Regulation of selenium metabolism and transport. Ann Rev Nutr. (2015) 35:109–34. 10.1146/annurev-nutr-071714-034250 25974694

[11] Chen J Berry MJ. Selenium and selenoproteins in the brain and brain diseases. J Neurochem. (2003) 86:1–12. 10.1046/j.1471-4159.2003.01854.x 12807419

[12] Takamura T. HepatokineSelenoprotein p-mediated reductive stress causes resistance to intracellular signal transduction. AntioxRedox Signal. (2020) 33:517–24. 10.1089/ars.2020.8087 32295394 PMC7409583

[13] Ogawa-Wong AN Berry MJ Seale LA. Selenum and metabolic disorders: an emphasis on type 2 diabetes riski. Nutrients. (2016) 8:80. 10.3390/nu8020080 26861388 PMC4772044

[14] Saito Y Takahashi K. Selenoprotein P: its structure and functions. J Health Sci. (2000) 46:409–13. 10.1248/jhs.46.409

[15] Olson GE Whitin JC Hill KE Winfrey VP Motley AK Austin LM. Extracellular glutathione peroxidase (Gpx3) binds specifically to basement membranes of mouse renal cortex tubule cells. Am J Physiol Renal Physiol. (2010) 298:F1244–53. 10.1152/ajprenal.00662.2009 20015939 PMC2867408

[16] Brodin O Hackler J Misra S Wendt S Sun Q Laaf E. Selenoprotein P as biomarker of selenium status in clinical trials with therapeutic dosages of selenite. Nutrients. (2020) 12:67. 10.3390/nu12041067 32290626 PMC7230801

[17] Kobayashi Y Ogra Y Ishiwata K Takayama H Aimi N Suzuki KT. Selenosugars are key and urinary metabolites for selenium excretion within the required to low-toxic range. Proc Nat Acad Sci USA. (2002) 99:15932–6. 10.1073/pnas.252610699 12441402 PMC138542

[18] Lee BJ Worland PJ Davis JN Stadtman TC Hatfield DL. Identification of a selenocysteyl-tRNA(Ser) in mammalian cells that recognizes the nonsense codon, UGA. J BiolChem. (1989) 264:9724–7. 10.1016/S0021-9258(18)81714-8 2498338

[19] Berry MJ Banu L Chen YY Mandel SJ Kieffer JD Harney JW. Recognition of UGA as a selenocysteine codon in type I deiodinase requires sequences in the 3' untranslated region. Nature. (1991) 353:273–6. 10.1038/353273a0 1832744

[20] Copeland PR Fletcher JE Carlson BA Hatfield DL Driscoll DM. A novel RNA binding protein, SBP2, is required for the translation of mammalian selenoprotein mRNAs. EMBO J. (2000) 19:306–14. 10.1093/emboj/19.2.306 10637234 PMC305564

[21] Hill KE Lloyd RS Burk RF. Conserved nucleotide sequences in the open reading frame and 3' untranslated region of selenoprotein P mRNA. Proc Nat Acad Sci USA. (1993) 90:537–41. 10.1073/pnas.90.2.537 8421687 PMC45698

[22] Saito Y Sato N Hirashima M Takebe G Nagasawa S Takahashi K. Domain structure of bi-functional selenoprotein P. Biochem J. (2004) 381:841–6. 10.1042/BJ20040328 15117283 PMC1133894

[23] Shetty S Copeland PR. Molecular mechanism of selenoprotein P synthesis. BiochimBiophysActa. (2018) 1862:2506–10. 10.1016/j.bbagen.2018.04.011 29656121 PMC6188828

[24] Takebe G Yarimizu J Saito Y Hayashi T Nakamura H Yodoi J. A comparative study on the hydroperoxide and thiol specificity of the glutathione peroxidase family and selenoprotein P. J BiolChem. (2002) 277:41254–8. 10.1074/jbc.M202773200 12185074

[25] Jones DP Carlson JL Mody VC Cai J Lynn MJ Sternberg P. Redox state of glutathione in human plasma. Free Rad BiolMed. (2000) 28:625–35. 10.1016/S0891-5849(99)00275-0 10719244

[26] Abdiu A Nakamura H Sahaf B Yodoi J Holmgren A Rosen A. Thioredoxin blood level increases after severe burn injury. AntioxRedox Signal. (2000) 2:707–16. 10.1089/ars.2000.2.4-707 11213476

[27] Takahashi K Akasaka M Yamamoto Y Kobayashi C Mizoguchi J Koyama J. Primary structure of human plasma glutathione peroxidase deduced from cDNA sequences. J Biochem. (1990) 108:145–8. 10.1093/oxfordjournals.jbchem.a123172 2229017

[28] Hatfield DL Tsuji PA Carlson BA Gladyshev VN. Selenium and selenocysteine: roles in cancer, health, and development. Trends Biochem Sci. (2014) 39:112–20. 10.1016/j.tibs.2013.12.007 24485058 PMC3943681

[29] Squires JE Berry MJ. Eukaryotic selenoprotein synthesis: mechanistic insight incorporating new factors and new functions for old factors. IUBMB Life. (2008) 60:232–5. 10.1002/iub.38 18344183

[30] Mihara H Kurihara T Watanabe T Yoshimura T Esaki N. cDNA cloning, purification, and characterization of mouse liver selenocysteine lyase. Candidate for selenium delivery protein in selenoprotein synthesis. J BiolChem. (2000) 275:6195–200. 10.1074/jbc.275.9.6195 10692412

[31] Schrauzer GN. Selenomethionine: a review of its nutritional significance, metabolism and toxicity. J Nutr. (2000) 130:1653–6. 10.1093/jn/130.7.1653 10867031

[32] Yan J Barrett JN. Purification from bovine serum of a survival-promoting factor for cultured central neurons and its identification as selenoprotein-P. J Neurosci. (1998) 18:8682–91. 10.1523/JNEUROSCI.18-21-08682.1998 9786975 PMC6793531

[33] Saito Y Yoshida Y Akazawa T Takahashi K Niki E. Cell death caused by selenium deficiency and protective effect of antioxidants. J BiolChem. (2003) 278:39428–34. 10.1074/jbc.M305542200 12888577

[34] Hill KE Zhou J Austin LM Motley AK Ham AJ Olson GE. The selenium-rich C-terminal domain of mouse selenoprotein P is necessary for the supply of selenium to brain and testis but not for the maintenance of whole body selenium. J BiologChem. (2007) 282:10972–80. 10.1074/jbc.M700436200 17311913

[35] Schomburg L Schweizer U Holtmann B Flohe L Sendtner M Kohrle J. Gene disruption discloses role of selenoprotein P in selenium delivery to target tissues. Biochem J. (2003) 370:397–402. 10.1042/bj20021853 12521380 PMC1223208

[36] Hill KE Zhou J McMahan WJ Motley AK Atkins JF Gesteland RF. Deletion of selenoprotein P alters distribution of selenium in the mouse. J BiolChem. (2003) 278:13640–6. 10.1074/jbc.M300755200 12574155

[37] Schweizer U Michaelis M Kohrle J Schomburg L. Efficient selenium transfer from mother to offspring in selenoprotein-P-deficient mice enables dose-dependent rescue of phenotypes associated with selenium deficiency. Biochem J. (2004) 378:21–6. 10.1042/bj20031795 14664694 PMC1223946

[38] Pietschmann N Rijntjes E Hoeg A Stoedter M Schweizer U Seemann P. Selenoprotein P is the essential selenium transporter for bones. Metallomics. (2014) 6:1043–9. 10.1039/C4MT00003J 24626785

[39] Burk RF Olson GE Hill KE Winfrey VP Motley AK Kurokawa S. Maternal-fetal transfer of selenium in the mouse. FASEB J. (2013) 27:3249–56. 10.1096/fj.13-231852 23651543 PMC3714584

[40] Olson GE Winfrey VP Nagdas SK Hill KE Burk RF. Apolipoprotein E receptor-2 (ApoER2) mediates selenium uptake from selenoprotein P by the mouse testis. J Biol Chem. (2007) 282:12290–7. 10.1074/jbc.M611403200 17314095

[41] Olson GE Winfrey VP Hill KE Burk RF. Megalin mediates selenoprotein P uptake by kidney proximal tubule epithelial cells. J BiolChem. (2008) 283:6854–60. 10.1074/jbc.M709945200 18174160

[42] Misu H Takayama H Saito Y Mita Y Kikuchi A Ishii KA. Deficiency of the hepatokineselenoprotein P increases responsiveness to exercise in mice through upregulation of reactive oxygen species and AMP-activated protein kinase in muscle. Nat Med. (2017) 23:508–16. 10.1038/nm.4295 28263310

[43] Chiu-Ugalde J Theilig F Behrends T Drebes J Sieland C Subbarayal P. Mutation of megalin leads to urinary loss of selenoprotein P and selenium deficiency in serum, liver, kidneys and brain. Biochem J. (2010) 431:103–11. 10.1042/BJ20100779 20653565

[44] Burk RF Hill KE Olson GE Weeber EJ Motley AK Winfrey VP. Deletion of apolipoprotein E receptor-2 in mice lowers brain selenium and causes severe neurological dysfunction and death when a low-selenium diet is fed. J Neurosci. (2007) 27:6207–11. 10.1523/JNEUROSCI.1153-07.2007 17553992 PMC6672153

[45] Kurokawa S Bellinger FP Hill KE Burk RF Berry MJ. Isoform-specific binding of selenoprotein P to the beta-propeller domain of apolipoprotein E receptor 2 mediates selenium supply. J BiologChem. (2014) 289:9195–207. 10.1074/jbc.M114.549014 24532792 PMC3979378

[46] Mita Y Nakayama K Inari S Nishito Y Yoshioka Y Sakai N. Selenoprotein P-neutralizing antibodies improve insulin secretion and glucose sensitivity in type 2 diabetes mouse models. Nat Commun. (2017) 8:1658. 10.1038/s41467-017-01863-z 29162828 PMC5698464

[47] Raman AV Pitts MW Seyedali A Hashimoto AC Seale LA Bellinger FP. Absence of selenoprotein P but not selenocysteine lyase results in severe neurological dysfunction. Genes Brain Behav. (2012) 11:601–13. 10.1111/j.1601-183X.2012.00794.x 22487427 PMC3389215

[48] Schweizer U Streckfuss F Pelt P Carlson BA Hatfield DL Kohrle J. Hepatically derived selenoprotein P is a key factor for kidney but not for brain selenium supply. Biochem J. (2005) 386:221–6. 10.1042/BJ20041973 15638810 PMC1134785

[49] Richardson DR. More roles for selenoprotein P: local selenium storage and recycling protein in the brain. Biochem J. (2005) 386:e5–7. 10.1042/BJ20050149 15720294 PMC1134806

[50] Renko K Werner M Renner-Muller I Cooper TG Yeung CH Hollenbach B. Hepatic selenoprotein P (SePP) expression restores selenium transport and prevents infertility and motor-incoordination in Sepp-knockout mice. Biochem J. (2008) 409:741–9. 10.1042/BJ20071172 17961124

[51] Schomburg L Schweizer U Kohrle J. Selenium and selenoproteins in mammals: extraordinary, essential, enigmatic. Cell Mol Life Sci. (2004) 61:1988–95. 10.1007/s00018-004-4114-z 15316649 PMC11138627

[52] Kikuchi N Satoh K Kurosawa R Yaoita N Elias-Al-Mamun M Siddique MAH. Selenoprotein P promotes the development of pulmonary arterial hypertension. Circulation. (2018) 138:600–23. 10.1161/CIRCULATIONAHA.117.033113 29636330

